# Personals

**Published:** 1918-08

**Authors:** 


### PERSONAL

Announcement of removal, travel, and other matters of interest are requested. Please report errors in the listing of any physician in the State and other directories, that we may co-operate with the State Society in securing a correct list.

Dr. Charles Haase of Elmira has recovered from an attack of Articular Rheumatism which laid him up for three months.

Drs. Lee Kinner, Carl Zimmerman, Arthur C. Glover and D. J. Tillou of Elmira have received commissions in the M. R. C. Dr. Zimmerman underwent an operation for hernia so that he would be accepted.

Dr. Louis J. Boyer announces the removal of his office and residence to 449 Delaware Ave. Practice limited to ear, nose and throat diseases.

Dr. C. Spencer Kinney delivered the graduation address to the hospital nurses of Middletown, N. Y. late in June. Dr. Kinney was for many years on the medical staff of the Middletown State Homeopathic Hospital. Since 1900 he has conducted the Eastern Sanitarium at Easton, Pa.

Dr. James Wright Putnam of Buffalo will serve until Sept. 1 in the military hospital at Williamsbridge.

Dr. Floyd Roberts of Lancaster was so unfortunate as to run down a little girl in Silver Springs, July 1. He was driving slowly and the child darted in front of his auto, from behind a line of parked cars, so that no blame attaches to him.

Capt. Elmer J. Preston of Corning, who was commissioned in the M. R. C. about a year ago, served for some months with British troops, later with the Am. Expeditionary Force. He was reported a German prisoner late in June.

